# Supplementary material for: Deep learning in MRI‐guided radiation therapy: A systematic review
Source: J Appl Clin Med Phys. 2023 Sep 15;25(2):e14155. doi: 10.1002/acm2.14155 (PMC10860468; doi:10.1002/acm2.14155)
Supplement: Supplementary file 1 — Supporting information [file ACM2-25-e14155-s001.docx]

**Tables**

**Table 1.** Literature Search and Yield as of 12/31/2022

| PubMed Search Terms | Category | Number of Studies |
| --- | --- | --- |
| "deep learning and (MRI or MR) and radiation therapy" | Image Segmentation | 78 |
|  | Image Synthesis | 81 |
|  | Radiomics | 24 |
|  | Real Time and 4D MRI | 14 |
|  | **Total from above** | 197 |
|  | Out of Scope | 138 |

**Supplemental Materials**

**Table 2.** Brain Segmentation Studies

| Study | Year | Target | Network  Architecture | Network  Features | Imaging Modalities | Patient Number | DSC |
| --- | --- | --- | --- | --- | --- | --- | --- |
| Simon *et al*^28^ | 2022 | AVM | U-Net |  | TOF MRA, T1WC, T2W | 23 | arteries: 0.86  veins: 0.91  brain: 0.98  CSF: 0.91 |
| Tian *et al*^67^ | 2022 | GBM | U-Net | 3D | T1C | 20 | 0.94 ± 0.012 |
| Bouget *et al*^131^ | 2022 | GBM | AGU-Net | Attention | T1W, FLAIR | 2134 | 0.86 ± 0.17 |
| Momin *et al*^64^ | 2022 | Glioma | Retina U-Net | BRATS  ROI  3D | T1, T1C, T2W, FLAIR | 369 | WT: 0.97 ± 0.03  TC: 0.90 ± 0.13  ET: 0.77 ± 0.22 |
| Ma *et al*^161^ | 2022 | Meningioma | CNN | Residual  Recurrent  Attention | T1C | 551 | 0.89 |
| Mi *et al*^162^ | 2022 | Temporis | U-Net |  | 3.0T T1C | 132 | 0.89 |
| Huang *et al*^65^ | 2022 | Tumor | DeepMedic | 3D | T1C | 176 | 0.81 |
| Chartrand  *et al*^66^ | 2022 | Tumor | U-Net | 3D | T1W | 530 | 2.5–6 mm: 0.68  > 10 mm: 0.86 |
| Yoo *et al*^35^ | 2022 | Tumor | U-Net | 2.5D | T1C | 65 | 0.75 |
| Ghaffari *et al*^68^ | 2022 | Tumor | Dense U-Net | Dense  3D | T1W, T1C, T2W, T2-FLAIR | 15 | WT: 0.83  TC: 0.77  ET: 0.60 |
| Bouget *et al*^163^ | 2021 | GBM | nnUNet | BRATS  3D | T1C, T1W | 1887 | 0.87 ± 0.15 |
| Pan *et al*^164^ | 2021 | HC | U-Net | Attention  Residual  ROI  3D | 1.5 T T1W | 235 | cohort b: 0.76 ± 0.04  cohort c: 0.80 ± 0.015 |
| Hsu *et al*^44^ | 2021 | Tumor | V-Net | Residual  3D | T1C, CECT | 511 | 0.76 ± 0.03 |
| Shirokikh  *et al*^165^ | 2021 | Tumor | U-Net | ROI | T1W | 1952 images | 0.64 ± 0.22 |
| Lin *et al*^166^ | 2021 | Tumor | U-Net | BRATS | T1, T1C, T2W,  T2-FLAIR | 369 | WT: 0.92 ± 0.05  TC: 0.89 ± 0.18  ET: 0.85 ± 0.17 |
| Huang *et al*^167^ | 2021 | Tumor | FCN | BRATS | T2W, T1C, T1, FLAIR | 384 | WT: 0.86  TC: 0.73  ET: 0.61 |
| Ahmadi *et al* ^34^ | 2021 | Tumor | QAIS-DSNN | BRATS | T1, T1C, T2W,  T2-FLAIR | 145 | WT: 0.92  ET: 0.75  TC: 0.80 |
| Lee *et al*^168^ | 2021 | Tumor | Dual Pathway  U-Net | 3D | 1.5 T T1C, T2W | 381 | 0.90 ± 0.05 |
| Eijgelaar  *et al*^169^ | 2020 | GBM | DeepMedic | BRATS,3D | T1W, T2W, T1C, FLAIR | 751 | BRATS: 0.80  Clinical: 0.49  Sparely Labeled: 0.67 |
| Rahmat  *et al*^169^ | 2020 | GBM | Deep Medic | 3D | 3.0 T DTI,  T2-FLAIR, T1C | 80 | 0.82 ± 0.17 |
| Ermis *et al*^170^ | 2020 | GBM | DenseNet | BRATS  Dense | T1W, T1C,  T2-FLAIR, T2W | 30 | WT: 0.83  TC: 0.81  ET: 0.81 |
| Tang *et al*^170^ | 2020 | Glioma | U-Net |  | CT, T1-FLAIR,  T2-FLAIR, T2W, T1C | 59 | 0.818 |
| Haensch *et al*^36^ | 2020 | HC | One Hundred  Layers  Tiramisu | Dense  2.5D | T1W | 45 | 0.67 |
| Mlynarski  *et al*^171^ | 2020 | Multi-Organ | U-Net |  | T1W | 44 | hippocampus: 0.88  pituitary: 0.80  brain: 0.99 |
| Zhou *et al*^172^ | 2020 | Tumor | FCN | ROI  2.5D | T1C | 934 | 0.81 ± 0.15 |
| Bousabarah *et al*^60^ | 2020 | Tumor | U-Net |  | 3.0 T T1C, T2W, T2-FLAIR | 509 | 0.60 |
| Xue et al^173^ | 2019 | Tumor | FCN |  | 3.0 T T1W | 1201 | 0.85 ± 0.08 |
| Charron  et al^174^ | 2018 | Tumor | DeepMedic | 3D | T1C, T2-FLAIR, T1W | 182 | 0.79 |
| Liu et al^22^ | 2017 | Tumor | DeepMedic | BRATS  3D | 3.0 T T1C | 240 | TC: 0.75 ± 0.07  ET: 0.81 ± 0.04 |

**Table 3.** H&N Segmentation Studies

| Study | Year | Target | Network  Architecture | Network  Features | Imaging  Modalities | Patient Number | DSC |
| --- | --- | --- | --- | --- | --- | --- | --- |
| Dai *et al*^175^ | 2022 | Multi-organ | MS R-CNN | Attention  Residual ROI  3D | 1.5T T1W | 60 | optic chiasm: 0.61 ± 0.14  oral cavity: 0.92 ± 0.07 |
| Tao *et al*^71^ | 2022 | NPC | SeqSeg | Attention  Residual  ROI  Recurrent | T1W, T2W,  T1C | 596 | 0.80 |
| Deng *et al*^73^ | 2022 | NPC | DenseNet,  V-Net | Dense  3D | 3.0T T1W, T2W, T1C  (separately) | 4478 | T1: 0.77±0.07  T2: 0.76±0.07 |
| Zhang *et al*^176^ | 2022 | NPC | AttR2U-Net | Attention | T1C | 93 | 0.82 |
| Outeiral *et al*^72^ | 2022 | Oropharyngeal  Cancer | U-Net | ROI  3D | T1W, T2W | 230 | 0.64 |
| Jiang et al^75^ | 2022 | Paratoid glands | GAN,  U-Net | Attention  Residual | 3.0T T2W, CT | 181 | right parotid gland: 0.81 ± 0.05  left parotid gland: 0.82 ± 0.03 |
| Kawahara  *et al*^177^ | 2022 | Paratoid glands,  Submandibular glands, lymph nodes | GAN | 2.5D | 1.5T T2W | 55 | right lymph node: 0.75  right parotid gland: 0.85 |
| Li *et al*^178^ | 2021 | NPC | DenseNet | Dense | T1W | 30 | 0.872 |
| Wahid *et al*^74^ | 2021 | Oropharyngeal  Cancer | Residual  U-Net | Residual  3D | 1.5 T T1W,  T2W, DCE, DWI | 30 | ALL MR Sequences:  0.71 ± 0.12  T1W + T2W:  0.73 ± 0.12 |
| Outeiral *et al*^179^ | 2021 | Oropharyngeal  Cancer | U-Net | 3D | T1W, T2W, T1C | 171 | 0.74 |
| Korte *et al*^180^ | 2021 | Paratoid glands,  Submandibular glands, lymph nodes | U-Net | ROI  3D | 1.5T T2W | 41 | LN Lvl IIIL:  0.56 ± 0.10  Left Parotid:  0.86 ± 0.07 |
| Ren *et al*^181^ | 2021 | Tumors | U-Net |  | PET, CT, T2W, T1W | 153 | 0.87 |
| Gurney-Champion  *et al*^182^ | 2020 | Lymph nodes | U-Net | 3D | 1.5 T DWI | 48 | 0.87 |
| Ke *et al*^183^ | 2020 | NPC | DenseNet | Dense  3D | 3.0 T T1W | 4100 | 0.77 ± 0.07 |
| Lin *et al*^184^ | 2019 | NPC | VoxResNet | Residual  3D | T1W, T2W, T1C, T1-Fat Suppressed | 203 | 0.79 |

**Table 4.** Abdomen, Heart, and Lung Segmentation Studies

| Study | Year | Target | Network  Architecture | Network  Features | Imaging  Modalities | Patient Number | DSC |
| --- | --- | --- | --- | --- | --- | --- | --- |
| Zhang  *et al*^176^ | 2022 | Multi-Organ | U-Net |  | 3T, T2w HASTE | 75 | DD: 0.88 ± 0.03  Stomach: 0.92 ± 0.02 |
| Ding  *et al*^79^ | 2022 | Multi-Organ | ResU-Net,  Active Contour Model | Residual  3D | 3T, T2w HASTE | 71 | DD: 0.49-0.69  Stomach: 0.56-0.77 |
| Luximon  *et al*^78^ | 2021 | Bowel Stomach | Dense U-Net | Dense | .35 T MRI | 116 | Bowel: 0.90 ± 0.04  Stomach: 0.91 ±0.02 |
| Morris *et al*^80^ | 2020 | Heart  substructures | U-Net | 3D | CT, T2W | 32 | Chambers:  0.88 ± 0.03  Great Vessels:  0.85 ± 0.03  pulmonary veins:  0.77 ± 0.04 |
| Chen *et al*^184^ | 2020 | Multi-Organ | Dense U-Net | Dense  2.5D | 3.0 T T1W VIBE | 102 | DD: 0.80 ± 0.07  Stomach: 0.92 ± 0.02 |
| Wang *et al*^81^ | 2019 | Lung Cancer | FCN |  | 3T T2W,  prior contours | 9 | 0.82 ± 0.10 |
| Wang *et al*^82^ | 2019 | Lung Cancer | CNN, GRU | Recurrent Attention | 3T T2W | 10 | Week 4: 0.78 ± 0.22  Week 5: 0.69 ± 0.24  Week 6: 0.69 ± 0.26 |
| Fu *et al*^184^ | 2018 | Multi-organ | Dense U-Net | Dense  3D | .35 T MRI | 120 | DD: 0.66 ± 0.09  Stomach: 0.85 ± 0.04 |

**Table 5.** Pelvic Segmentation Studies

| Study | Year | Target | Network  Architecture | Network  Features | Imaging  Modalities | Patient  Number | DSC |
| --- | --- | --- | --- | --- | --- | --- | --- |
| Groendahl *et al*^185^ | 2022 | Anal Cancer | U-Net | ROI | 3.0 T2W, DWI, PET/CT,  contrast CT | 36 | PET, contrast CT:  0.83 ± 0.08  PET, contrast CT, T2W:  0.81 ± 0.08 |
| Shaaer *et al*^86^ | 2022 | Catheters  (Cervix) | U-Net |  | 1.5 T T1W, T2W | 20 | 0.59 ± 0.10 |
| Zabihollahy *et al*^87^ | 2022 | Cervical Cancer | U-Net | 3D | 1.5T T2W MRI | 123 | 0.85 ± 0.03 |
| Cao *et al*^186^ | 2022 | Cervical Cancer | Dual-path CNN | Residual | T2W, CT | 65 | Small: 0.65 ± 0.03  Medium: 0.79 ± 0.02  Large: 0.75 ± 0.04 |
| Yoganathan *et al*^187^ | 2022 | Cervical Cancer  Multi-Organ | ResNet50,  InceptionResNetV2 | Residual  2.5D | 1.5T T1W | 39 | GTV: 0.62 ± 0.14 |
| Breto *et al*^188^ | 2022 | Cervical Cancer  Multi-Organ | Mask R-CNN | Residual | 0.35T  MRIdian | 15 | GTV: 0.67 ± 0.30 |
| Li *et al^189^* | 2022 | Liver,  Kidney,  Cervical Cancer | nnU-Net |  | T2W | 6 | Liver GTV: 0.94 ± 0.01  Kidney GTV: 0.95 ± 0.02  Cervix GTV: 0.97 ± 0.02 |
| Fransson *et al*^190^ | 2022 | Prostate and OARs | U-Net | ROI | 3T, T2W | 17 | CTV: 0.92 ± 0.03  Bladder: 0.93 ± 0.07  Rectum: 0.84 ± 0.10 |
| Eidex *et al*^61^ | 2022 | Prostate Cancer | Mask R-CNN | Residual  ROI  3D | T1W | 77 | Prostate: 0.90 ± 0.09  DIL: 0.84 ± 0.12 |
| Li *et al*^191^ | 2021 | Anal Cancer | U-Net | Attention | Not Specified | 304 | 0.98 |
| Huang *et al*^192^ | 2021 | Colorectal  Cancer | RU-Net | ROI  3D | T2W | 64 | 0.76 |
| Zabihollahy *et al*^193^ | 2021 | Female Bladder, Rectum,  Sigmoid  Colon | 3D U-Net  3D Dense U-Net | Dense  ROI  3D | 1.5 T T2W | 129, 52 | Bladder: 0.94 ± 0.05  Rectum: 0.88 ± 0.04 Sigmoid: 0.80 ± 0.05 |
| Cha *et al*^186^ | 2021 | Prostate | DeepLabV3 + | Residual | 3.0 T T2W, sCT | 50 | Prostate: 0.89  Bladder: 0.99 |
| Comelli *et al*^194^ | 2021 | Prostate | E-Net |  | T1W | 85 | 0.91 |
| Savenije *et al*^195^ | 2020 | Bladder,  Rectum, Femur | Deep Medic | 3D | 3.0 T T1W | 150 | Bladder: 0.96 ± 0.02  Rectum: 0.88 ± 0.05  femurs: 0.97 ±0.01 |
| Dai *et a*l^196^ | 2020 | Catheters  (prostate) | AGU-Net | Attention  3D | 1.5 T T2W | 20 | Average displacement:  0.37±1.68 mm |
| Singhrao  *et al*^89^ | 2020 | Fiducials  (prostate) | pix2pix (GAN) |  | T1W | 56 | 0.67 |
| Gustafsson *et al*^132^ | 2020 | Fiducials  (prostate) | HighRes3DNet | Residual  3D | 3T T2W | 326 | 0.98 ± 0.002 |
| Sanders *et al*^197^ | 2020 | Prostate | DenseNet-201 |  | T1W, T2W, T1C | 200 | Prostate: 0.90 ± 0.04  Bladder: 0.91 ± 0.06  Rectum: 0.96 ± 0.04 |
| da Silva  *et al*^198^ | 2020 | Prostate | Hybrid atlas,  active contour |  | T2W | 56 | 0.85 |
| Chen *et al*^199^ | 2020 | Prostate cancer | MB-U-Net |  | 3.0 T T2W, ADC, DWI | 136 | 0.63 |
| Zaffino *et a*l^200^ | 2019 | Catheters  (Cervix) | U-Net | 3D | T2W | 50 | 0.60 ± 0.17 |
| Yang *et al*^201^ | 2019 | Prostate | MICS-Net |  | T2W, CT | 22 | 0.83 ± 0.04 |
| Elguindi  *et al*^202^ | 2019 | Prostate and OARs | Deep LabV3+ | Residual | T2W | 50 | CTV: 0.83 ± 0.06  Bladder: 0.93 ± 0.04  Rectum: 0.82 ± 0.05 |
| Nie *et al*^88^ | 2019 | Prostate and OARS | STRAINet (GAN) | Residual | 3.0 T T1W | 35 | Prostate: 0.91 ± 0.01  Bladder: 0.97 ± 0.01  Rectum: 0.91 ± 0.03 |
| Feng *et al*^203^ | 2018 | Prostate and OARs | ResNet | Residual | Not specified | 40 | Prostate: 0.90 ± 0.02  Bladder: 0.96 ± 0.01  Rectum: 0.89 ± 0.03 |
| Wang  *et al*^37^ | 2018 | Rectal  Cancer | U-Net | 2.5D | 3.0T T2W | 93 | 0.74 ± 0.14 |

**Table 6.** MRI-based Synthetic CT Studies for Photon Therapy

| Study | Year | Site | Network  Architecture | Network  Features | GAN Type | Imaging  Modalities | Patient  Number | MAE | Dosimetry |
| --- | --- | --- | --- | --- | --- | --- | --- | --- | --- |
| Ranjan  *et al*^204^ | 2022 | Brain | U-Net |  | pix2pix | T2W | 18 | 0.03 ± 0.02 | N/A |
| Wang *et al*^205^ | 2022 | Brain | U-Net | 3D | pix2pix | 1.5T T1W | 31 | MSE:  0.12 ± 0.04 % | N/A |
| Jabbarpour  *et al*^206^ | 2022 | Brain | FCN | Residual | cycleGAN | 3T T1W, T2W (separately) | 189 | 61.9 ± 22.6 HU | 2%/2 mm:  95.0 ± 3.7% |
| Scholey *et al*^207^ | 2022 | H&N | U-Net | 3D | N/A | 1.5T T1W | 120 | whole body:  93.3 ± 27.5  soft tissue:  78.2 ± 27.5  bone:  138.0 ± 43.4  (HU) | 2%/2 mm: 96.8 ± 2.6% |
| Florkow *et al*^208^ | 2022 | Hip  Pelvis | U-Net | 3D | N/A | 3.0 T T1W | 30 | Femur: 23 ± 24  Pelvis:  -15 ± 29  (Mean Error HU) | N/A |
| Li *et al*^209^ | 2022 | Liver | U-Net |  | N/A | .35T sim | 37 | 35.6 HU | N/A |
| Lenkowicz *et al*^210^ | 2022 | Lung | U-Net |  | pix2pix | .35T sim | 60 | 54.9 ± 10.5 HU | 2%/2mm:  96.1 ± 5.1% |
| Reaunga-mornrat  *et al*^113^ | 2022 | Pelvis | FCN | Residual | relativistic GAN | High and low res  Dixon MRI | 45 | Median  normalized mutual  information:  1.28 | N/A |
| O’Connor *et al*^211^ | 2022 | Pelvis | U-Net |  | cGAN | 4.0 T T1 VIBE Dixon | 40 | whole body:  34.7 ± 5.1  bone:  109.4 ± 12.3  soft tissue:  25.2 ± 3.4 (HU) | 3%/2 mm: 99.8% |
| Tsekas *et al*^117^ | 2022 | Abdomen  Torso | U-Net | 3D | N/A | 1.5 T1W | 124 | speed:  1.5 seconds  per segment | 2% / 2 mm:  96.3% ± 4.2%  (for deep learning dose calculation) |
| Zhao *et al*^114^ | 2022 | Pelvis | Transformer-CNN hybrid | Residual Transformer | cGAN | T2W | 19 | 45.1 HU | N/A |
| Hsu *et al*^212^ | 2022 | Prostate | U-Net | 2.5D | pix2pix | .35T sim | 57 | pelvis:  30.1 ± 4.2  soft tissue:  19.6 ± 2.3  bone:  158.5 ± 26.0 (HU) | 2%/2 mm: 99.9% |
| Olber *et al*^213^ | 2021 | Abdomen | Dense U-Net | Dense | GAN | .35T sim | 89 | No gas:  90 ± 29 HU  Gas:  143 ± 29 HU | 3%/3 mm:  well matched:  98.3 ± 1.3%,  poorly matched: 93.9 ± 9.8% |
| Kang *et al*^214^ | 2021 | Abdomen  Pelvis  Thorax | U-Net | Residual  2.5D | cycleGAN | .35T sim | 90 | 59.2 ± 5.8 HU | 2%/ 2mm,  10% LDT: >97% |
| Lerner *et al*^215^ | 2021 | Brain | FCN | 3D | N/A | Dixon MRI | 20 | Body: 62.2 ± 4.1 Brain: 9.5 ± 0.7 Bone: 173.8 ± 18.2 | 2%/ 2 mm: 99.8 ± 0.2 |
| Yuan *et al*^102^ | 2021 | Brain | Res U-Net | Residual | N/A | 1.5T T1W | 30 | 86.6 ± 34.1 HU | D95 Difference: 1.1% |
| Liu *et al*^216^ | 2021 | Brain | ResNet | Residual | GAN | T1W | 12 | N/A | 2%/2mm:  99.9 ± 0.2% |
| Koerkamp *et al*^217^ | 2021 | Breast | Not  Specified |  | revGAN | 1.5T T1W | 39 | 106 HU | 2%/2 mm,  10% LDT: 99.4% |
| Baydoun *et al*^218^ | 2021 | Cervix | U-Net |  | cGAN | T2W | 11 | 115.74 ± 21.84 HU | N/A |
| Olin *et al*^219^ | 2021 | H&N | U-Net | 3D | N/A | Dixon MRI | 6,17 | External:  78 ± 13 HU  Local:  76 ± 12 HU | 2%/ 2mm:  98.8 ± 0.8% |
| Liu *et al*^220^ | 2021 | H&N | Z-Net, FCN |  | CycleGAN | Dixon MRI | 164 | 0.04 | N/A |
| Touati *et al*^221^ | 2021 | H&N | U-Net |  | CycleGAN | 3.0T T1W | 56 | 45.3 ± 1.9 HU | N/A |
| Song *et al*^222^ | 2021 | NPC | U-Net |  | N/A | 1.5T T1W | 35 | 125.6 HU | 3%/2mm:  97.7 ± 0.7% |
| Ma *et al*^223^ | 2021 | NPC | U-Net |  | pix2pix | 3.0T T1W | 20 | 102.6 ± 11.4 HU | 2 mm/3%,  10% LDT:  99.1% ± 0.3% |
| Szalkowski *et al*^224^ | 2021 | Pelvis | FCN | 3D | GAN | T2W | 11 | 72.9 ± 88.1 HU | N/A |
| Boni *et al*^109^ | 2021 | Pelvis | Not  Specified |  | conditional  CycleGAN | 1.5T, 3.0T T2W | 38 | 59.8 HU | 2%/ 2mm:  95.5 ± 2.2% |
| Bird *et al*^225^ | 2021 | Pelvis  (ano-rectal) | U-Net |  | pix2pix | 1.5T T2W | 90 | 35.1 ± 7.9 HU | 2%/ 2mm:  99.8 ± 0.1% |
| Yoo *et al*^226^ | 2021 | Prostate | FCN | Residual | cycleGAN | T2W | 113 | 96.95 ± 10.32 HU | 2%/ 2mm:  93.9 ± 3.2% |
| Farjam *et al*^227^ | 2021 | Prostate | U-Net |  | N/A | .35T sim | 30 | whole body:  29.7 ± 4.4,  fat:  16.34 ± 2.67  muscle:  23.36 ± 2.85  bone:  105.90 ± 22.80 (HU) | N/A |
| Cusumano *et al*^228^ | 2020 | Abdomen  Pelvis | U-Net |  | pix2pix | .35T sim | 120 | Abdomen:  78.7 ± 18.5  Pelvis:  54.3 ± 11.9 (HU) | abdomen 2%/2 mm:  98.7 ± 1.1%  pelvis 2%/2 mm:  99.0 ± 0.7% |
| Liu *et al*^229^ | 2020 | Abdomen | U-Net |  | N/A | Dixon MRI | 31 | liver: 24.1  spleen: 28.6  lungs: 105.7  vertebral bodies: 110.1 (HU) | mean differences for all PTV and OAR dose metrics  < .15 Gy |
| Massa *et al*^110^ | 2020 | Brain | Inception V3, U-Net |  | N/A | 1.5T T1W, T2W, T1C, FLAIR  (separately) | 92 | 51.2 ± 4.5 HU | N/A |
| Andres *et al*^230^ | 2020 | Brain | HighResNet | Residual  3D | N/A | 1.5T, 3.0T T1W, T1C | 402 | 92 ± 23 HU | 3%/3 mm:  99.8 ± 0.2% |
| Koike *et al*^111^ | 2020 | Brain (GMB) | U-Net |  | pix2pix | T1W, T2W, FLAIR | 15 | whole body:  108.1 ± 24.0  soft tissue:  38.9 ± 10.7  bone:  366.2 ± 62.0 (HU) | 2%/ 2mm: 99.2 ± 1.0% |
| Olin *et al*^231^ | 2020 | H&N | U-Net |  | N/A | Dixon MRI | 11 | Body: 94 ± 14  Air: 300 ± 69  Soft tissue:  41 ± 4  bone:  258 ± 51 (HU) | all dosimetric  parameters  within ±1% |
| Largent *et al*^101^ | 2020 | H&N | U-Net |  | GAN | T2W | 8 | 82.8 HU | N/A |
| Qi *et al*^232^ | 2020 | H&N | U-Net |  | pix2pix | T1W, T2W,  T1C, Dixon | 45 | 70.0 ± 12.0 HU | 2%/ 2mm: 99.3 ± 0.2 |
| Klages *et al*^233^ | 2020 | H&N | CycleGAN |  | GAN | mDixon FFE | 23 | pix2pix: 66.9±7.3  CycleGAN: 82.3±6.4 (HU) | Absolute percent mean/max dose errors < 2% |
| Tie *et al*^234^ | 2020 | H&N  (nasopharynx) | ResU-Net | Residual | cGAN | T1W, T1C, T2W | 32 | 75.7 ± 14.6  bone:  194.6 ± 38.9 (HU) | N/A |
| Bahrami *et al*^235^ | 2020 | Pelvis | SegNet  (U-Net) | Residual | N/A | 3.0T, T2W | 15 | 30.0 ± 10.4  HU | N/A |
| Florkow *et al*^236^ | 2020 | Pelvis | U-Net | 3D | N/A | 3T T1W | 23,  17 dogs | humans: 33  dogs: 35 (HU) | N/A |
| Kazemirfar *et al*^237^ | 2019 | Brain | U-Net |  | GAN | 1.5T T1Gd | 77 | 47.2 ± 11.0 HU | 2%/ 2mm:  99.2 ± .8% |
| Lei *et al*^238^ | 2019 | Brain  Prostate | FCN | Dense | CycleGAN | Brain: T1W  Prostate: T2W | 44 | Brain: 55.7  Prostate: 50.8 (HU) | N/A |
| Liu *et al*^239^ | 2019 | Brain | VGG16 | Residual | N/A | 1.5T T1W | 40 | 75 ± 23 HU | The absolute  percentage  differences:  PTV: 0.24 ± 0.46%  max dose: 1.39 ± 1.31%  V95: 0.27 ± 0.79% |
| Liu *et al*^108^ | 2019 | Liver | FCN | Dense | cycleGAN | T1W | 21 | 72.9 ± 18.2 HU | 2%/ 2 mm  10% LDT:  97.0 ± 2.9% |
| Olberg *et al*^240^ | 2019 | Breast | FCN |  | GAN | .35T sim | 60 | 16.1 ± 3.5 HU | 2%/ 2mm > 98% |
| Gupta *et al*^241^ | 2019 | H&N | U-Net |  | N/A | 3T Dixon | 60 | 81.0 ± 14.6  air:  233.8 ± 28.0  soft tissue:  17.6 ± 3.4  bone:  193.1 ± 38.3 (HU) | mean target dose difference of  2.3 ± 0.1% |
| Dinkla *et al*^112^ | 2019 | H&N | U-Net |  | N/A | 3T T2W | 34 | 75 ± 9 HU | 2%/2mm:  95.6 ± 2.9% |
| Wang *et al*^242^ | 2019 | NPC | U-Net |  | N/A | 1.5T T2W | 33 | whole body:  131 ± 24  soft tissue:  97 ± 13  bone:  357 ± 44 (HU) | N/A |
| Fu *et al*^243^ | 2019 | Pelvis | U-Net | 3D | N/A | 1.5T T1W | 20 | 2D CNN:  40.5 ± 5.4  3D CNN:  37.6 ± 5.1 (HU) | N/A |
| Largent *et al*^244^ | 2019 | Prostate | U-Net |  | GAN | 3T T2W | 39 | U-Net:  34.4 ± 7.7  GAN:  34.1 ± 7.5 (HU) | 1% /1 mm,  10% LDT:  99.2 ± 1.0 |
| Emami *et al*^245^ | 2018 | Brain | ResNet | Residual | GAN | 1.0T T1Gd | 15 | whole body:  89.3 ± 10.3  tissue:  41.9 ± 8.6  Bone/Air:  240-255 (HU) | N/A |
| Arabi *et al*^246^ | 2018 | Pelvis | U-Net |  | N/A | 3T T2W | 39 | 32.7 ± 7.9 HU | 1%/1 mm:  94.6 ± 5.7% |
| Chen *et al*^247^ | 2018 | Prostate | U-Net |  | N/A | 3T T2W | 51 | 30.0 ± 4.9 HU | 2%/2 mm: 99.4% |
| Han^248^ | 2017 | Brain | U-Net |  | N/A | 1.5T T1W | 18 | 84.8 ± 17.3 HU | N/A |

**Table 7**. MRI-based Synthetic CT Studies for Proton and Boron Therapy

| Study | Year | Site | Network  Architecture | Network  Features | GAN | Imaging Modalities | Patient Number | MAE | Dosimetry |
| --- | --- | --- | --- | --- | --- | --- | --- | --- | --- |
| Zimmermann  *et al*^98^ | 2022 | Brain | Res U-Net | Residual | N/A | T1W, T2W, T1C | 47 | T1:  body 79.8  bone 216.3  T2:  body 71.1  bone 186.1  T1C:  body 82.9  bone 236.4 (HU) | dose parameters within 1% |
| Zhao *et al*^118^ | 2022 | Brain | SARU | Attention Residual  Boron- therapy | N/A | T1W | 104 | Head: 67.8 ± 24.3 Skull: 144.0 ± 45.83 Brain: 14.9 ± 21.2 (HU) | 2%/2 mm: 0.98 ± 0.01 |
| Wang *et al*^6^ | 2022 | Brain | Res U-Net | Residual sRPSP | ccGAN  (constant  cycle) | T1W, T2W, FLAIR | 195 | 42 ± 13   HU | 10%/3 mm: 55-60% from chart |
| Wang *et al*^249^ | 2021 | Brain | Attention  U-Net | Attention | cycleGAN | 1.5T, 3.0T T1W | 125 | 65.3 ± 13.9 HU | mean absolute differences:  V95 1.1 ± 0.8%  80% beam axis distal falloff  1.1 ± 0.9 mm |
| Liu *et al*^115^ | 2021 | H&N | Residual FCN | Residual, Dual Energy CT | label GAN (conditional cycleGAN) | 1.5T T1W | 57 | Low Energy CT:  80.0 ± 18.1 High Energy CT: 80.2 ± 16.3 (HU) | N/A |
| Maspero  *et al*^116^ | 2020 | Brain | U-Net | 2.5D | cGAN | 1.5T, 3.0T T1W | 60 | 61 ± 14 HU | 2%/2mm: photon 99.5 ± 0.8%  proton 99.2 ± 1.1% |
| Kazemifar  *et al*^250^ | 2020 | Brain, proton | U-Net |  | GAN | 1.5T T1W | 77 | 47.2 ± 11.0 HU | mean absolute difference:  CTV < .5% (0.3 Gy)  OAR < 2% (1.2 Gy) |
| Florkow *et al*^251^ | 2020 | Wilms Tumor | U-Net | 3D | N/A | 1.5T T1W, T2W | 54 | 57 ± 12 HU | 2%/2 mm: VMAT >99%  PBS (pencil beam scanning) >96% |
| Liu *et al*^252^ | 2019 | Liver | FCN | Dense | CycleGAN | T1W | 21 | 72.9 ± 18.2 HU | 1%/1 mm: >99% |
| Shafai-Erfani  et al^253^ | 2019 | Brain | FCN | Dense | cycleGAN | 1.5 T1W | 50 | 54.6 ± 6.8 HU | 2%/ 2 mm, 10% LDT: 98% |
| Neppi *et al*^254^ | 2019 | Brain | U-Net | 3D | N/A | 1.5T T1W | 89 | 137 ± 32 HU | 2%/ 2mm: 99.3% |
| Liu *et al*^255^ | 2019 | Pelvis | FCN | Dense | cycleGAN | 1.5 T2W | 17 | 51.3 ± 16.9 HU | 2 mm/2%: 97.95 ± 2.95%  mean Bragg peak shift:  0.18 ± 0.07 cm |

**Table 8.** Synthetic MRI Studies

| Study | Year | Site | Network  Architecture | Network  Features | GAN | Input  Modality | Output  Modality | Patient  Number | Results |
| --- | --- | --- | --- | --- | --- | --- | --- | --- | --- |
| Dai *et al*^120^ | 2021 | H&N | MS-RCNN | Attention  Dense  ROI  3D | N/A | CT | T1W | 108 | local DSC 0.77  public DSC: 0.86 |
| Kieselmann  *et al*^9^ | 2021 | H&N | U-Net |  | CycleGAN | CT | 3T T2W | 27 | DSC: 0.77±0.07 |
| Gotoh  *et al*^256^ | 2021 | Lumbar Spine | U-Net |  | pix2pix | CT | 3T T2W | 22 | PSNR:  18.4 ± 2.1  MSE:  8876.7 ± 1192.9 |
| Kalantar  *et al*^257^ | 2021 | Pelvis | U-Net |  | CycleGAN | CT | 1.5T T1W | 17 | PSNR:  18.3 ± 0.2  MAE:  0.057 ± 0.001 |
| Lei *et al*^121^ | 2021 | Pelvis | FCN | Attention  Dense | CycleGAN | CT | T2W | 140 | DSC: 0.95 ± 0.05 |
| Xu *et al*^122^ | 2020 | Brain | FCN | Dense  3D | N/A | CT | T1W | 391 | sMRI MAE: 15.5  sCT MAE: 9.1 |
| Li *et al*^99^ | 2020 | Brain | U-Net | Attention Dense | N/A | CT | 1.5T T1W | 34 | MAE: 74.2  PSNR: 32.4 |
| Fu *et al*^123^ | 2020 | Pelvis | U-Net | Attention  Dense  3D | CycleGAN | CBCT | T2W | 100 | bladder 0.96 ± 0.03  prostate 0.91 ± 0.08  rectum: 0.93 ± 0.04  (DSC) |
| Lei *et al*^8^ | 2020 | Pelvis | FCN | Attention  Dense  3D | CycleGAN | CBCT | T2W | 100 | bladder 0.95 ± 0.02  prostate 0.86 ± 0.06  rectum 0.91 ± 0.04  (DSC) |
| Dong *et al*^7^ | 2019 | Pelvis | U-Net | 3D | CycleGAN | CT | T2W | 102 | Bladder: 0.95±0.03  Prostate: 0.87±0.04  Rectum: 0.89±0.04  (DSC) |

**Table 9.** Intramodal MR Synthesis Studies

| Study | Year | Site | Network  Architecture | Network Features | GAN | Input | Output | Patients | Results |
| --- | --- | --- | --- | --- | --- | --- | --- | --- | --- |
| Xie et al^128^ | 2022 | brain  (BraTS) | ResUnet | Residual | parallel  CycleGANs | 1x1x3 mm^3^  T1W, T2W, T1C, FLAIR (separately) | 1x1x1 mm^3^  T1W, T2W,  T1C, FLAIR  (separately) | 300 | SSIM: 0.98 ± 0.01 |
| Xie et al^258^ | 2022 | brain  (BraTS) | Retina U-Net | ROI | N/A | T1W | T1C | 369 | SSIM: 0.99 ± 0.01 |
| Zhou *et al*^10^ | 2022 | brain | Dual path DenseNet | Dense | GAN | 3.0T T2-Flair | 4x High Res | 237 | DSC: .79 |
| Chen *et al*^125^ | 2022 | pelvis | ResUnet | Residual | CycleGAN | T1W | Rotated T1W | 23 | Prone MAE:  35.6 ± 4.0  Lateral MAE:  40.5 ± 5.8 |
| Zormpas-Petridis *et al*^126^ | 2022 | prostate,  mesothelioma | ResUnet |  | N/A | ADC map | ADC Uncertainty Map | 44 | ADC uncertainty differed by 4.3% for the prostate and 3.7% for mesothelioma |
| Preetha *et al*^12^ | 2021 | brain | U-Net |  | pix2pix | T1W, T2W, FLAIR | Synthetic Contrast | 206 | median SSIM: 0.82 |
| Chun *et al*^11^ | 2019 | torso  abdomen | FCN | Residual | N/A | .35T MRI |  | 480 | SSIM: 0.96 |

**Table 10.** Cancer Detection and Staging Studies.

| Study | Year | Purpose | Site | Architecture | Network  Features | Input Modality | Patient  Number | Results |
| --- | --- | --- | --- | --- | --- | --- | --- | --- |
| Yang *et al*^13^ | 2022 | false positive  segmentation reduction | Brain | Siamese network, SVM | Residual | T1C | 242 | AUC: 0.93 |
| Liang *et al*^259^ | 2022 | NPC Staging | Brain | FCN | Attention  Residual | T1C | 320 | AUC: 0.88 |
| Gustafsson *et al*^260^ | 2022 | prostate RT DICOM structure classification | Prostate | InceptionResNetV2 | Residual | sCT | 40 | F1: 0.985 |
| Chakrabarty *et al*^133^ | 2021 | brain tumor  classification | Brain | CNN | BRATS  3D | T1C | 2105 | Internal:  0.85-100  External: 0.73-0.99  (AUC) |
| Gao *et al*^140^ | 2020 | Tumor Recurrence or Necrosis | Brain | CNN |  | T1W, T1C, T2W | 146 | AUC: 0.96 |
| Zhang *et al*^261^ | 2020 | suspected lesion  classification | Brain | Faster R-CNN, RUSBooster |  | T1W | 121 | AUC: 0.79 |
| Zhou *et al*^262^ | 2020 | brain metastases  classification | Brain | CNN |  | T1W | 266 | sensitivity: 0.81 |
| Chen *et al*^139^ | 2019 | cerebrial microbleeds  classification | Brain | ResNet | Residual  3D | 7T TOF,  TOF-SWI MRI | 73 | AUC: 0.97 |

**Table 11.** Treatment Response Studies

| Study | Year | Purpose | Site | Network  Architecture | Network Features | Input  Modality | Patient  Number | Results |
| --- | --- | --- | --- | --- | --- | --- | --- | --- |
| Huisman *et al*^143^ | 2022 | Post-radiation brain aging rate | Brain | FCN |  | 3T T1W | 32 | accelerated aging rate:  2.78 years/year |
| Keek *et al*^15^ | 2022 | adverse reaction prediction | Brain | xception, xgboost |  | T1Gd | 1641 | AUC: 0.71  recall: 0.80 |
| Jalalifar *et al*^145^ | 2022 | local tumor control prediction | Brain | InceptionResNet + LSTM + Clinical Feature Fusion | Residual Recurrent | T1Gd, T2-Flair | 124 | AUC: 0.86 |
| Jalalifar  *et al*^146^ | 2022 | Local metastases treatment  response | Brain | Hybrid CNN-Transformer | 3D  Transformer  Residual | T1W, T2-FLAIR | 124 | AUC: 0.91 |
| Hua  *et al*^144^ | 2022 | Distant Metastases Prediction | H&N | xception |  | 1.5T T1W | 441 | AUC: 0.88 |
| Tomita  *et al*^263^ | 2022 | laryngeal and hypopharyngeal cancer local recurrence prediction | H&N | xception |  | 1.5T DWI | 70 | AUC: 0.77 |
| Ottens  *et al*^264^ | 2022 | DCE-MRI hysiological parameter estimation for tracer-kinetic  modeling | Pancreas | GRU | Recurrent | 3T DCE-MRI | 28 | random error reduced by  factor of 4.8 |
| Zhu  *et al*^14^ | 2022 | rectal cancer treatment response | Rectum | CNN |  | 3T DWI | 472 | AUC: 0.93 |
| Zhang  *et al*^265^ | 2021 | Distant Metastases Prediction | H&N | ResNet, clinical, regression | Residual | T2W, T1C | 189 | AUC: 0.80 |
| Jing  *et al*^142^ | 2021 | NPC Risk Score Prediction | H&N | DenseNet + clinical data | Dense  3D | T1W,T2C, clinical data | 1846 | C-index: 0.67 |
| Jang  *et al*^266^ | 2021 | rectal cancer pathological response | Rectum | ShuffleNet, LSTM | Recurrent | T2W | 466 | pCR: 0.76  Good Response: 0.72 (AUC) |
| Jin *et al*^267^ | 2021 | treatment response | Rectum | CNN | 3D | T1W,T2W, T1C,DWI | 622 | cohort 1: 0.95  cohort 2: 0.92  (AUC) |
| Gao  *et al*^268^ | 2021 | sarcoma response prediction | Whole Body | VGG-19 |  | .35T DWI | 35 | accuracy: 0.83 |
| Metz  *et al*^269^ | 2020 | free water correction for Glioblastoma Recurrence prediction | Brain | ANN |  | DTI | 35 | AUC: 0.90 |
| Zhang  *et al*^270^ | 2020 | rectal cancer treatment response prediction | Rectum | CNN |  | DKI, 3.0T T2W | 401 | pCR: 0.99  treatment response:  0.70  Tumor downstaging:  0.79 (AUC) |
| Fu *et al*^271^ | 2020 | rectal cancer treatment response prediction | Rectum | LASSO, VGG19 |  | ADC | 43 | AUC: 0.73 |

**Table 12.** Real Time and 4D MRI Studies.

| Study | Year | Site | Purpose | Network  Architecture | Network  Features | Inputs | Output | Patient  Number | Results |
| --- | --- | --- | --- | --- | --- | --- | --- | --- | --- |
| Gulamhussene *et al*^17^ | 2022 | liver | 4D | U-Net |  | 2D cine MRI | 4D MRI | 20 | target registration error: 1.2 ± 0.7mm |
| Xiao *et at*^272^ | 2022 | liver | 4D | U-Net | 3D | 3D MRI, 4D MRI | High quality 4D MR | 39 | inference time: 69.3 ± 5.9 ms  Anterior-Posterior ROI  tracking error:  0.50 ± 0.55 |
| Driever *et al*^155^ | 2022 | Stomach | Location Probability | U-Net |  | 2D T2W  Coronal MRI | 3D  iso-probability surfaces | 18 | Median Standard Deviation:  organ deformation:  2.0-2.9 mm  respiratory deformation:  2.7-8.8 mm |
| Nie *et al*^152^ | 2022 | lung | Real Time MRI | auto-regression |  | 2D cine MRI | 4D MRI | 8 | Displacement at 8 Hz:  autoregression 0.06 ± 0.02 mm  LSTM 0.18 ± 0.06 mm |
| Shao *et al*^16^ | 2022 | heart liver | Real Time MRI | FCN |  | k-space  trajectory, prior^16^ MRI, undersampled cine MRI | 3D MRI | 8 cardiac  9 liver | 13-spoke k-space cardiac DSC:  0.89 ± 0.02 |
| Tamura *et al*^273^ | 2022 | lung | Real Time MRI | CycleGAN |  | 2D cine MRI | 4DCT | 5 | 3D motion predicted 1.5 seconds in future |
| Wei *et al*^274^ | 2022 | liver | Real Time MRI | FCN |  | T1W Planning MRI,  undersampled  treatment MRI | Treatment MRI | 3 | With 12.5% radial undersampling and 15% increase in noise,  SNR improved 4.46dB and  SSIM by 28% |
| Frueh *et al*^18^ | 2022 | Abdomen heart | Real Time MRI | CNN |  | 2D CINE MRI | Local affinity matrices,  segmentation | 1190 (MRI) | Liver DSC: 0.95/0.96  left ventricle DSC: 0.89/0.90  (forward pass/backwards pass): |
| Grandinetti  *et al*^275^ | 2022 | liver | Real Time MRI | CNN |  | Planning Dixon MRI,  undersampled MRI | Reconstructed MRI | 3 | With 12.5% radial undersampling, PSNR: 34.4 |
| Zormpas-Petridis *et al*^276^ | 2021 | prostate lung | acquisition time  reduction | U-Net |  | subsampled DWI | DWI | 39 | PSNR: 55.7 |
| Terpstra *et al*^153^ | 2021 | lung | Real Time MRI | FCN | 3D | 3D cine MRI | DVF | 27 | target registration error: 1.87 ± 1.65 mm |
| Romaguera  *et al*^154^ | 2020 | liver | Real Time MRI | FCN | Residual Recurrent | 3T T2W | Next slice | 85 | vessel position median accuracy:  0.45 mm |
| Terpstra *et al*^277^ | 2020 | abdomen | Real Time MRI | FCN |  | 1.5T 2D cine MRI | DVF | 135 | Standard Reconstruction with  undersampling factor of 25:  Standard method SSIM: 0.82 ± 0.07 Deep Learning SSIM: 0.80 ± 0.08 |
| Kim *et al*^127^ | 2019 | torso  abdomen | Real Time  MRI | FCN | Residual | .35T MRI | Higher spatial,  temporal  resolution | 4 | SSIM: 0.89 |
