# Supplementary material for: Deep learning in MRI‐guided radiation therapy: A systematic review
Source: J Appl Clin Med Phys. 2023 Sep 15;25(2):e14155. doi: 10.1002/acm2.14155 (PMC10860468; doi:10.1002/acm2.14155)
Supplement: Supplementary file 2 — Supporting information [file ACM2-25-e14155-s002.docx]

**References**

161. Ma X, Zhao Y, Lu Y, et al. A dual-branch hybrid dilated CNN model for the AI-assisted segmentation of meningiomas in MR images [published online ahead of print 20221109]. *Comput Biol Med.* 2022;151(Pt A):106279.

162. Mi E, Mauricaite R, Pakzad-Shahabi L, Chen J, Ho A, Williams M. Deep learning-based quantification of temporalis muscle has prognostic value in patients with glioblastoma [published online ahead of print 20211130]. *Br J Cancer.* 2022;126(2):196-203.

163. Bouget D, Eijgelaar RS, Pedersen A, et al. Glioblastoma Surgery Imaging-Reporting and Data System: Validation and Performance of the Automated Segmentation Task [published online ahead of print 20210917]. *Cancers (Basel).* 2021;13(18).

164. Pan K, Zhao L, Gu S, et al. Deep learning-based automatic delineation of the hippocampus by MRI: geometric and dosimetric evaluation [published online ahead of print 20210114]. *Radiat Oncol.* 2021;16(1):12.

165. Shirokikh B, Shevtsov A, Dalechina A, et al. Accelerating 3D Medical Image Segmentation by Adaptive Small-Scale Target Localization [published online ahead of print 20210213]. *J Imaging.* 2021;7(2).

166. Lin M, Momin S, Lei Y, et al. Fully automated segmentation of brain tumor from multiparametric MRI using 3D context deep supervised U-Net [published online ahead of print 20210711]. *Med Phys.* 2021;48(8):4365-4374.

167. Huang D, Wang M, Zhang L, Li H, Ye M, Li A. Learning rich features with hybrid loss for brain tumor segmentation [published online ahead of print 20210730]. *BMC Med Inform Decis Mak.* 2021;21(Suppl 2):63.

168. Lee CC, Lee WK, Wu CC, et al. Applying artificial intelligence to longitudinal imaging analysis of vestibular schwannoma following radiosurgery [published online ahead of print 20210204]. *Sci Rep.* 2021;11(1):3106.

169. Eijgelaar RS, Visser M, Müller DMJ, et al. Robust Deep Learning-based Segmentation of Glioblastoma on Routine Clinical MRI Scans Using Sparsified Training [published online ahead of print 20200930]. *Radiol Artif Intell.* 2020;2(5):e190103.

170. Tang F, Liang S, Zhong T, et al. Postoperative glioma segmentation in CT image using deep feature fusion model guided by multi-sequence MRIs [published online ahead of print 20191024]. *Eur Radiol.* 2020;30(2):823-832.

171. Mlynarski P, Delingette H, Alghamdi H, Bondiau PY, Ayache N. Anatomically consistent CNN-based segmentation of organs-at-risk in cranial radiotherapy [published online ahead of print 20200213]. *J Med Imaging (Bellingham).* 2020;7(1):014502.

172. Zhou Z, Sanders JW, Johnson JM, et al. MetNet: Computer-aided segmentation of brain metastases in post-contrast T1-weighted magnetic resonance imaging [published online ahead of print 20200913]. *Radiother Oncol.* 2020;153:189-196.

173. Xue J, Wang B, Ming Y, et al. Deep learning-based detection and segmentation-assisted management of brain metastases. *Neuro Oncol.* 2020;22(4):505-514.

174. Charron O, Lallement A, Jarnet D, Noblet V, Clavier JB, Meyer P. Automatic detection and segmentation of brain metastases on multimodal MR images with a deep convolutional neural network [published online ahead of print 20180209]. *Comput Biol Med.* 2018;95:43-54.

175. Dai X, Lei Y, Wang T, et al. Multi-organ auto-delineation in head-and-neck MRI for radiation therapy using regional convolutional neural network [published online ahead of print 20220121]. *Phys Med Biol.* 2022;67(2).

176. Zhang Y, Liang Y, Ding J, et al. A Prior Knowledge-Guided, Deep Learning-Based Semiautomatic Segmentation for Complex Anatomy on Magnetic Resonance Imaging [published online ahead of print 20220604]. *Int J Radiat Oncol Biol Phys.* 2022;114(2):349-359.

177. Kawahara D, Tsuneda M, Ozawa S, et al. Deep learning-based auto segmentation using generative adversarial network on magnetic resonance images obtained for head and neck cancer patients [published online ahead of print 20220309]. *J Appl Clin Med Phys.* 2022;23(5):e13579.

178. Li Y, Han G, Liu X. DCNet: Densely Connected Deep Convolutional Encoder-Decoder Network for Nasopharyngeal Carcinoma Segmentation [published online ahead of print 20211126]. *Sensors (Basel).* 2021;21(23).

179. Rodríguez Outeiral R, Bos P, Al-Mamgani A, Jasperse B, Simões R, van der Heide UA. Oropharyngeal primary tumor segmentation for radiotherapy planning on magnetic resonance imaging using deep learning [published online ahead of print 20210702]. *Phys Imaging Radiat Oncol.* 2021;19:39-44.

180. Korte JC, Hardcastle N, Ng SP, Clark B, Kron T, Jackson P. Cascaded deep learning-based auto-segmentation for head and neck cancer patients: Organs at risk on T2-weighted magnetic resonance imaging [published online ahead of print 20211101]. *Med Phys.* 2021;48(12):7757-7772.

181. Ren J, Eriksen JG, Nijkamp J, Korreman SS. Comparing different CT, PET and MRI multi-modality image combinations for deep learning-based head and neck tumor segmentation [published online ahead of print 20210715]. *Acta Oncol.* 2021;60(11):1399-1406.

182. Gurney-Champion OJ, Kieselmann JP, Wong KH, Ng-Cheng-Hin B, Harrington K, Oelfke U. A convolutional neural network for contouring metastatic lymph nodes on diffusion-weighted magnetic resonance images for assessment of radiotherapy response. *Phys Imaging Radiat Oncol.* 2020;15:1-7.

183. Ke L, Deng Y, Xia W, et al. Development of a self-constrained 3D DenseNet model in automatic detection and segmentation of nasopharyngeal carcinoma using magnetic resonance images [published online ahead of print 20200629]. *Oral Oncol.* 2020;110:104862.

184. Lin L, Dou Q, Jin YM, et al. Deep Learning for Automated Contouring of Primary Tumor Volumes by MRI for Nasopharyngeal Carcinoma [published online ahead of print 20190326]. *Radiology.* 2019;291(3):677-686.

185. Groendahl AR, Moe YM, Kaushal CK, et al. Deep learning-based automatic delineation of anal cancer gross tumour volume: a multimodality comparison of CT, PET and MRI [published online ahead of print 20211116]. *Acta Oncol.* 2022;61(1):89-96.

186. Cha E, Elguindi S, Onochie I, et al. Clinical implementation of deep learning contour autosegmentation for prostate radiotherapy [published online ahead of print 20210303]. *Radiother Oncol.* 2021;159:1-7.

187. Yoganathan SA, Paul SN, Paloor S, et al. Automatic segmentation of magnetic resonance images for high-dose-rate cervical cancer brachytherapy using deep learning [published online ahead of print 20220209]. *Med Phys.* 2022;49(3):1571-1584.

188. Breto AL, Spieler B, Zavala-Romero O, et al. Deep Learning for Per-Fraction Automatic Segmentation of Gross Tumor Volume (GTV) and Organs at Risk (OARs) in Adaptive Radiotherapy of Cervical Cancer [published online ahead of print 20220518]. *Front Oncol.* 2022;12:854349.

189. Li Z, Zhang W, Li B, et al. Patient-specific daily updated deep learning auto-segmentation for MRI-guided adaptive radiotherapy [published online ahead of print 20221112]. *Radiother Oncol.* 2022;177:222-230.

190. Fransson S, Tilly D, Strand R. Patient specific deep learning based segmentation for magnetic resonance guided prostate radiotherapy [published online ahead of print 20220603]. *Phys Imaging Radiat Oncol.* 2022;23:38-42.

191. Li D, Chu X, Cui Y, Zhao J, Zhang K, Yang X. Improved U-Net based on contour prediction for efficient segmentation of rectal cancer [published online ahead of print 20211024]. *Comput Methods Programs Biomed.* 2022;213:106493.

192. Huang YJ, Dou Q, Wang ZX, et al. 3-D RoI-Aware U-Net for Accurate and Efficient Colorectal Tumor Segmentation [published online ahead of print 20211109]. *IEEE Trans Cybern.* 2021;51(11):5397-5408.

193. Zabihollahy F, Viswanathan AN, Schmidt EJ, Morcos M, Lee J. Fully automated multiorgan segmentation of female pelvic magnetic resonance images with coarse-to-fine convolutional neural network [published online ahead of print 20211021]. *Med Phys.* 2021;48(11):7028-7042.

194. Comelli A, Dahiya N, Stefano A, et al. Deep Learning-Based Methods for Prostate Segmentation in Magnetic Resonance Imaging [published online ahead of print 20210115]. *Appl Sci (Basel).* 2021;11(2).

195. Savenije MHF, Maspero M, Sikkes GG, et al. Clinical implementation of MRI-based organs-at-risk auto-segmentation with convolutional networks for prostate radiotherapy [published online ahead of print 20200511]. *Radiat Oncol.* 2020;15(1):104.

196. Dai X, Lei Y, Zhang Y, et al. Automatic multi‐catheter detection using deeply supervised convolutional neural network in MRI‐guided HDR prostate brachytherapy. *Medical Physics.* 2020;47(9):4115-4124.

197. Sanders JW, Lewis GD, Thames HD, et al. Machine Segmentation of Pelvic Anatomy in MRI-Assisted Radiosurgery (MARS) for Prostate Cancer Brachytherapy [published online ahead of print 20200704]. *Int J Radiat Oncol Biol Phys.* 2020;108(5):1292-1303.

198. da Silva GLF, Diniz PS, Ferreira JL, et al. Superpixel-based deep convolutional neural networks and active contour model for automatic prostate segmentation on 3D MRI scans [published online ahead of print 20200621]. *Med Biol Eng Comput.* 2020;58(9):1947-1964.

199. Chen Y, Ruan D, Xiao J, et al. Fully automated multiorgan segmentation in abdominal magnetic resonance imaging with deep neural networks [published online ahead of print 20200830]. *Med Phys.* 2020;47(10):4971-4982.

200. Zaffino P, Pernelle G, Mastmeyer A, et al. Fully automatic catheter segmentation in MRI with 3D convolutional neural networks: application to MRI-guided gynecologic brachytherapy [published online ahead of print 20190814]. *Phys Med Biol.* 2019;64(16):165008.

201. Yang W, Shi Y, Park SH, Yang M, Gao Y, Shen D. An Effective MR-Guided CT Network Training for Segmenting Prostate in CT Images [published online ahead of print 20191216]. *IEEE J Biomed Health Inform.* 2020;24(8):2278-2291.

202. Elguindi S, Zelefsky MJ, Jiang J, et al. Deep learning-based auto-segmentation of targets and organs-at-risk for magnetic resonance imaging only planning of prostate radiotherapy [published online ahead of print 20191212]. *Phys Imaging Radiat Oncol.* 2019;12:80-86.

203. Feng Z, Nie D, Wang L, Shen D. SEMI-SUPERVISED LEARNING FOR PELVIC MR IMAGE SEGMENTATION BASED ON MULTI-TASK RESIDUAL FULLY CONVOLUTIONAL NETWORKS [published online ahead of print 20180524]. *Proc IEEE Int Symp Biomed Imaging.* 2018;2018:885-888.

204. Ranjan A, Lalwani D, Misra R. GAN for synthesizing CT from T2-weighted MRI data towards MR-guided radiation treatment [published online ahead of print 20211106]. *Magma.* 2022;35(3):449-457.

205. Wang CC, Wu PH, Lin G, et al. Magnetic Resonance-Based Synthetic Computed Tomography Using Generative Adversarial Networks for Intracranial Tumor Radiotherapy Treatment Planning [published online ahead of print 20220226]. *J Pers Med.* 2022;12(3).

206. Jabbarpour A, Mahdavi SR, Vafaei Sadr A, Esmaili G, Shiri I, Zaidi H. Unsupervised pseudo CT generation using heterogenous multicentric CT/MR images and CycleGAN: Dosimetric assessment for 3D conformal radiotherapy [published online ahead of print 20220131]. *Comput Biol Med.* 2022;143:105277.

207. Scholey JE, Rajagopal A, Vasquez EG, Sudhyadhom A, Larson PEZ. Generation of synthetic megavoltage CT for MRI-only radiotherapy treatment planning using a 3D deep convolutional neural network [published online ahead of print 20220808]. *Med Phys.* 2022;49(10):6622-6634.

208. Florkow MC, Willemsen K, Zijlstra F, et al. MRI-based synthetic CT shows equivalence to conventional CT for the morphological assessment of the hip joint [published online ahead of print 20210712]. *J Orthop Res.* 2022;40(4):954-964.

209. Li W, Kazemifar S, Bai T, et al. Synthesizing CT images from MR images with deep learning: model generalization for different datasets through transfer learning [published online ahead of print 20210224]. *Biomed Phys Eng Express.* 2021;7(2).

210. Lenkowicz J, Votta C, Nardini M, et al. A deep learning approach to generate synthetic CT in low field MR-guided radiotherapy for lung cases [published online ahead of print 20220905]. *Radiother Oncol.* 2022;176:31-38.

211. O'Connor LM, Choi JH, Dowling JA, Warren-Forward H, Martin J, Greer PB. Comparison of Synthetic Computed Tomography Generation Methods, Incorporating Male and Female Anatomical Differences, for Magnetic Resonance Imaging-Only Definitive Pelvic Radiotherapy [published online ahead of print 20220208]. *Front Oncol.* 2022;12:822687.

212. Hsu SH, Han Z, Leeman JE, Hu YH, Mak RH, Sudhyadhom A. Synthetic CT generation for MRI-guided adaptive radiotherapy in prostate cancer [published online ahead of print 20220923]. *Front Oncol.* 2022;12:969463.

213. Olberg S, Chun J, Su Choi B, et al. Abdominal synthetic CT reconstruction with intensity projection prior for MRI-only adaptive radiotherapy [published online ahead of print 20211001]. *Phys Med Biol.* 2021;66(20).

214. Kang SK, An HJ, Jin H, et al. Synthetic CT generation from weakly paired MR images using cycle-consistent GAN for MR-guided radiotherapy [published online ahead of print 20210619]. *Biomed Eng Lett.* 2021;11(3):263-271.

215. Lerner M, Medin J, Jamtheim Gustafsson C, Alkner S, Siversson C, Olsson LE. Clinical validation of a commercially available deep learning software for synthetic CT generation for brain [published online ahead of print 20210407]. *Radiat Oncol.* 2021;16(1):66.

216. Liu X, Emami H, Nejad-Davarani SP, et al. Performance of deep learning synthetic CTs for MR-only brain radiation therapy [published online ahead of print 20210107]. *J Appl Clin Med Phys.* 2021;22(1):308-317.

217. Groot Koerkamp ML, de Hond YJM, Maspero M, et al. Synthetic CT for single-fraction neoadjuvant partial breast irradiation on an MRI-linac [published online ahead of print 20210416]. *Phys Med Biol.* 2021;66(8).

218. Baydoun A, Xu KE, Heo JU, et al. Synthetic CT Generation of the Pelvis in Patients With Cervical Cancer: A Single Input Approach Using Generative Adversarial Network [published online ahead of print 20210108]. *IEEE Access.* 2021;9:17208-17221.

219. Olin AB, Thomas C, Hansen AE, et al. Robustness and Generalizability of Deep Learning Synthetic Computed Tomography for Positron Emission Tomography/Magnetic Resonance Imaging-Based Radiation Therapy Planning of Patients With Head and Neck Cancer [published online ahead of print 20210726]. *Adv Radiat Oncol.* 2021;6(6):100762.

220. Liu Y, Chen A, Shi H, et al. CT synthesis from MRI using multi-cycle GAN for head-and-neck radiation therapy [published online ahead of print 20210626]. *Comput Med Imaging Graph.* 2021;91:101953.

221. Touati R, Le WT, Kadoury S. A feature invariant generative adversarial network for head and neck MRI/CT image synthesis [published online ahead of print 20210423]. *Phys Med Biol.* 2021;66(9).

222. Song L, Li Y, Dong G, et al. Artificial intelligence-based bone-enhanced magnetic resonance image-a computed tomography/magnetic resonance image composite image modality in nasopharyngeal carcinoma radiotherapy. *Quant Imaging Med Surg.* 2021;11(12):4709-4720.

223. Ma X, Chen X, Li J, Wang Y, Men K, Dai J. MRI-Only Radiotherapy Planning for Nasopharyngeal Carcinoma Using Deep Learning [published online ahead of print 20210908]. *Front Oncol.* 2021;11:713617.

224. Szalkowski G, Nie D, Zhu T, Yap PT, Lian J. Synthetic digital reconstructed radiographs for MR-only robotic stereotactic radiation therapy: A proof of concept [published online ahead of print 20211004]. *Comput Biol Med.* 2021;138:104917.

225. Bird D, Nix MG, McCallum H, et al. Multicentre, deep learning, synthetic-CT generation for ano-rectal MR-only radiotherapy treatment planning [published online ahead of print 20201129]. *Radiother Oncol.* 2021;156:23-28.

226. Yoo GS, Luu HM, Kim H, et al. Feasibility of Synthetic Computed Tomography Images Generated from Magnetic Resonance Imaging Scans Using Various Deep Learning Methods in the Planning of Radiation Therapy for Prostate Cancer [published online ahead of print 20211223]. *Cancers (Basel).* 2021;14(1).

227. Farjam R, Nagar H, Kathy Zhou X, Ouellette D, Chiara Formenti S, DeWyngaert JK. Deep learning-based synthetic CT generation for MR-only radiotherapy of prostate cancer patients with 0.35T MRI linear accelerator [published online ahead of print 20210628]. *J Appl Clin Med Phys.* 2021;22(8):93-104.

228. Cusumano D, Lenkowicz J, Votta C, et al. A deep learning approach to generate synthetic CT in low field MR-guided adaptive radiotherapy for abdominal and pelvic cases [published online ahead of print 20201017]. *Radiother Oncol.* 2020;153:205-212.

229. Liu L, Johansson A, Cao Y, Dow J, Lawrence TS, Balter JM. Abdominal synthetic CT generation from MR Dixon images using a U-net trained with 'semi-synthetic' CT data [published online ahead of print 20200615]. *Phys Med Biol.* 2020;65(12):125001.

230. Arends SRS, Savenije MHF, Eppinga WSC, van der Velden JM, van den Berg CAT, Verhoeff JJC. Clinical utility of convolutional neural networks for treatment planning in radiotherapy for spinal metastases [published online ahead of print 20220217]. *Phys Imaging Radiat Oncol.* 2022;21:42-47.

231. Olin AB, Hansen AE, Rasmussen JH, et al. Feasibility of Multiparametric Positron Emission Tomography/Magnetic Resonance Imaging as a One-Stop Shop for Radiation Therapy Planning for Patients with Head and Neck Cancer [published online ahead of print 20200716]. *Int J Radiat Oncol Biol Phys.* 2020;108(5):1329-1338.

232. Qi M, Li Y, Wu A, et al. Multi-sequence MR image-based synthetic CT generation using a generative adversarial network for head and neck MRI-only radiotherapy [published online ahead of print 20200226]. *Med Phys.* 2020;47(4):1880-1894.

233. Klages P, Benslimane I, Riyahi S, et al. Patch-based generative adversarial neural network models for head and neck MR-only planning [published online ahead of print 20191225]. *Med Phys.* 2020;47(2):626-642.

234. Tie X, Lam SK, Zhang Y, Lee KH, Au KH, Cai J. Pseudo-CT generation from multi-parametric MRI using a novel multi-channel multi-path conditional generative adversarial network for nasopharyngeal carcinoma patients [published online ahead of print 20200221]. *Med Phys.* 2020;47(4):1750-1762.

235. Bahrami A, Karimian A, Fatemizadeh E, Arabi H, Zaidi H. A new deep convolutional neural network design with efficient learning capability: Application to CT image synthesis from MRI [published online ahead of print 20200906]. *Med Phys.* 2020;47(10):5158-5171.

236. Florkow MC, Zijlstra F, Willemsen K, et al. Deep learning-based MR-to-CT synthesis: The influence of varying gradient echo-based MR images as input channels [published online ahead of print 20191008]. *Magn Reson Med.* 2020;83(4):1429-1441.

237. Kazemifar S, McGuire S, Timmerman R, et al. MRI-only brain radiotherapy: Assessing the dosimetric accuracy of synthetic CT images generated using a deep learning approach [published online ahead of print 20190411]. *Radiother Oncol.* 2019;136:56-63.

238. Lei Y, Harms J, Wang T, et al. MRI-only based synthetic CT generation using dense cycle consistent generative adversarial networks [published online ahead of print 20190612]. *Med Phys.* 2019;46(8):3565-3581.

239. Liu F, Yadav P, Baschnagel AM, McMillan AB. MR-based treatment planning in radiation therapy using a deep learning approach. *J Appl Clin Med Phys.* 2019;20(3):105-114.

240. Olberg S, Zhang H, Kennedy WR, et al. Synthetic CT reconstruction using a deep spatial pyramid convolutional framework for MR-only breast radiotherapy [published online ahead of print 20190807]. *Med Phys.* 2019;46(9):4135-4147.

241. Gupta D, Kim M, Vineberg KA, Balter JM. Generation of Synthetic CT Images From MRI for Treatment Planning and Patient Positioning Using a 3-Channel U-Net Trained on Sagittal Images [published online ahead of print 20190925]. *Front Oncol.* 2019;9:964.

242. Wang Y, Liu C, Zhang X, Deng W. Synthetic CT Generation Based on T2 Weighted MRI of Nasopharyngeal Carcinoma (NPC) Using a Deep Convolutional Neural Network (DCNN) [published online ahead of print 20191129]. *Front Oncol.* 2019;9:1333.

243. Fu J, Yang Y, Singhrao K, et al. Deep learning approaches using 2D and 3D convolutional neural networks for generating male pelvic synthetic computed tomography from magnetic resonance imaging [published online ahead of print 20190726]. *Med Phys.* 2019;46(9):3788-3798.

244. Largent A, Barateau A, Nunes JC, et al. Comparison of Deep Learning-Based and Patch-Based Methods for Pseudo-CT Generation in MRI-Based Prostate Dose Planning [published online ahead of print 20190907]. *Int J Radiat Oncol Biol Phys.* 2019;105(5):1137-1150.

245. Emami H, Dong M, Nejad-Davarani SP, Glide-Hurst CK. Generating synthetic CTs from magnetic resonance images using generative adversarial networks [published online ahead of print 20180614]. *Med Phys.* 2018. doi: 10.1002/mp.13047.

246. Arabi H, Dowling JA, Burgos N, et al. Comparative study of algorithms for synthetic CT generation from MRI: Consequences for MRI-guided radiation planning in the pelvic region [published online ahead of print 20181010]. *Med Phys.* 2018;45(11):5218-5233.

247. Chen S, Qin A, Zhou D, Yan D. Technical Note: U-net-generated synthetic CT images for magnetic resonance imaging-only prostate intensity-modulated radiation therapy treatment planning [published online ahead of print 20181113]. *Med Phys.* 2018;45(12):5659-5665.

248. Han X. MR-based synthetic CT generation using a deep convolutional neural network method [published online ahead of print 20170321]. *Med Phys.* 2017;44(4):1408-1419.

249. Wang C, Uh J, Merchant TE, Hua CH, Acharya S. Facilitating MR-Guided Adaptive Proton Therapy in Children Using Deep Learning-Based Synthetic CT [published online ahead of print 20210625]. *Int J Part Ther.* 2022;8(3):11-20.

250. Kazemifar S, Barragán Montero AM, Souris K, et al. Dosimetric evaluation of synthetic CT generated with GANs for MRI-only proton therapy treatment planning of brain tumors [published online ahead of print 20200326]. *J Appl Clin Med Phys.* 2020;21(5):76-86.

251. Florkow MC, Guerreiro F, Zijlstra F, et al. Deep learning-enabled MRI-only photon and proton therapy treatment planning for paediatric abdominal tumours [published online ahead of print 20201007]. *Radiother Oncol.* 2020;153:220-227.

252. Liu Y, Lei Y, Wang Y, et al. MRI-based treatment planning for proton radiotherapy: dosimetric validation of a deep learning-based liver synthetic CT generation method [published online ahead of print 20190716]. *Phys Med Biol.* 2019;64(14):145015.

253. Shafai-Erfani G, Lei Y, Liu Y, et al. MRI-Based Proton Treatment Planning for Base of Skull Tumors [published online ahead of print 20190930]. *Int J Part Ther.* 2019;6(2):12-25.

254. Neppl S, Landry G, Kurz C, et al. Evaluation of proton and photon dose distributions recalculated on 2D and 3D Unet-generated pseudoCTs from T1-weighted MR head scans [published online ahead of print 20190704]. *Acta Oncol.* 2019;58(10):1429-1434.

255. Liu Y, Lei Y, Wang Y, et al. Evaluation of a deep learning-based pelvic synthetic CT generation technique for MRI-based prostate proton treatment planning [published online ahead of print 20191021]. *Phys Med Biol.* 2019;64(20):205022.

256. Gotoh M, Nakaura T, Funama Y, et al. Virtual magnetic resonance lumbar spine images generated from computed tomography images using conditional generative adversarial networks [published online ahead of print 20211110]. *Radiography (Lond).* 2022;28(2):447-453.

257. Kalantar R, Lin G, Winfield JM, et al. Automatic Segmentation of Pelvic Cancers Using Deep Learning: State-of-the-Art Approaches and Challenges [published online ahead of print 20211022]. *Diagnostics (Basel).* 2021;11(11).

258. Xie H, Lei Y, Wang T, et al. Magnetic resonance imaging contrast enhancement synthesis using cascade networks with local supervision. *Medical Physics.* 2022;49(5):3278-3287.

259. Liang S, Dong X, Yang K, et al. A multi-perspective information aggregation network for automatedT-staging detection of nasopharyngeal carcinoma [published online ahead of print 20221209]. *Phys Med Biol.* 2022;67(24).

260. Jamtheim Gustafsson C, Lempart M, Swärd J, et al. Deep learning-based classification and structure name standardization for organ at risk and target delineations in prostate cancer radiotherapy [published online ahead of print 20211008]. *J Appl Clin Med Phys.* 2021;22(12):51-63.

261. Zhang M, Young GS, Chen H, et al. Deep-Learning Detection of Cancer Metastases to the Brain on MRI [published online ahead of print 20200313]. *J Magn Reson Imaging.* 2020;52(4):1227-1236.

262. Zhou Z, Sanders JW, Johnson JM, et al. Computer-aided Detection of Brain Metastases in T1-weighted MRI for Stereotactic Radiosurgery Using Deep Learning Single-Shot Detectors [published online ahead of print 20200317]. *Radiology.* 2020;295(2):407-415.

263. Tomita H, Kobayashi T, Takaya E, et al. Deep learning approach of diffusion-weighted imaging as an outcome predictor in laryngeal and hypopharyngeal cancer patients with radiotherapy-related curative treatment: a preliminary study [published online ahead of print 20220224]. *Eur Radiol.* 2022;32(8):5353-5361.

264. Ottens T, Barbieri S, Orton MR, et al. Deep learning DCE-MRI parameter estimation: Application in pancreatic cancer [published online ahead of print 20220607]. *Med Image Anal.* 2022;80:102512.

265. Zhang L, Wu X, Liu J, et al. MRI-Based Deep-Learning Model for Distant Metastasis-Free Survival in Locoregionally Advanced Nasopharyngeal Carcinoma [published online ahead of print 20200809]. *J Magn Reson Imaging.* 2021;53(1):167-178.

266. Jang BS, Lim YJ, Song C, et al. Image-based deep learning model for predicting pathological response in rectal cancer using post-chemoradiotherapy magnetic resonance imaging [published online ahead of print 20210615]. *Radiother Oncol.* 2021;161:183-190.

267. Jin C, Yu H, Ke J, et al. Predicting treatment response from longitudinal images using multi-task deep learning [published online ahead of print 20210325]. *Nat Commun.* 2021;12(1):1851.

268. Gao Y, Ghodrati V, Kalbasi A, et al. Prediction of soft tissue sarcoma response to radiotherapy using longitudinal diffusion MRI and a deep neural network with generative adversarial network-based data augmentation [published online ahead of print 20210514]. *Med Phys.* 2021;48(6):3262-3372.

269. Metz MC, Molina-Romero M, Lipkova J, et al. Predicting Glioblastoma Recurrence from Preoperative MR Scans Using Fractional-Anisotropy Maps with Free-Water Suppression [published online ahead of print 20200319]. *Cancers (Basel).* 2020;12(3).

270. Zhang XY, Wang L, Zhu HT, et al. Predicting Rectal Cancer Response to Neoadjuvant Chemoradiotherapy Using Deep Learning of Diffusion Kurtosis MRI [published online ahead of print 20200421]. *Radiology.* 2020;296(1):56-64.

271. Fu J, Zhong X, Li N, et al. Deep learning-based radiomic features for improving neoadjuvant chemoradiation response prediction in locally advanced rectal cancer [published online ahead of print 20200402]. *Phys Med Biol.* 2020;65(7):075001.

272. Xiao H, Ni R, Zhi S, et al. A dual-supervised deformation estimation model (DDEM) for constructing ultra-quality 4D-MRI based on a commercial low-quality 4D-MRI for liver cancer radiation therapy [published online ahead of print 20220225]. *Med Phys.* 2022;49(5):3159-3170.

273. Tamura Y, Demachi K, Igaki H, Okamoto H, Nakano M. A Real-Time Four-Dimensional Reconstruction Algorithm of Cine-Magnetic Resonance Imaging (Cine-MRI) Using Deep Learning [published online ahead of print 20220303]. *Cureus.* 2022;14(3):e22826.

274. Wei R, Chen J, Liang B, Chen X, Men K, Dai J. Real-time 3D MRI reconstruction from cine-MRI using unsupervised network in MRI-guided radiotherapy for liver cancer [published online ahead of print 20221212]. *Med Phys.* 2022. doi: 10.1002/mp.16141.

275. Grandinetti J, Gao Y, Gonzalez Y, Deng J, Shen C, Jia X. MR image reconstruction from undersampled data for image-guided radiation therapy using a patient-specific deep manifold image prior [published online ahead of print 20221121]. *Front Oncol.* 2022;12:1013783.

276. Zormpas-Petridis K, Tunariu N, Curcean A, et al. Accelerating Whole-Body Diffusion-weighted MRI with Deep Learning-based Denoising Image Filters [published online ahead of print 20210714]. *Radiol Artif Intell.* 2021;3(5):e200279.

277. Terpstra ML, Maspero M, d'Agata F, et al. Deep learning-based image reconstruction and motion estimation from undersampled radial k-space for real-time MRI-guided radiotherapy [published online ahead of print 20200807]. *Phys Med Biol.* 2020;65(15):155015.
